# Association Between Preoperative Nerve Conduction Study Severity and Outcomes After Open Carpal Tunnel Release

**DOI:** 10.7759/cureus.117641

**Published:** 2026-10-08

**Authors:** Nay Aung Zin, Mohamed Arsham Abdul Rasheed, Izka Mohamed, Ko Ko Kyaw, Thu Ya Aung, De Nyein Lwin, Thi La Si Thu

**Affiliations:** 1 Orthopedics and Traumatology Department, Kulhudhuffushi Regional Hospital, Kulhudhuffushi, MDV; 2 Orthopedic Surgery Department, Kulhudhuffushi Regional Hospital, Kulhudhuffushi, MDV; 3 Medicine Department, Kulhudhuffushi Regional Hospital, Kulhudhuffushi, MDV; 4 Critical Care Unit (CCU), Indira Gandhi Memorial Hospital (IGMH), Malé, MDV; 5 Orthopedics and Traumatology Department, University of Medicine 1, Yangon, Yangon, MMR; 6 Orthopedics Department, University of Medicine 2, Yangon, Yangon, MMR; 7 Orthopedics and Trauma Department, Yangon Orthopaedic Hospital, Yangon, MMR

**Keywords:** boston carpal tunnel questionnaire, carpal tunnel release, carpal tunnel syndrome, electrophysiological severity, nerve conduction study

## Abstract

Background

Carpal tunnel syndrome (CTS) is a common upper-extremity entrapment neuropathy for which nerve conduction studies (NCS) are widely used to confirm median neuropathy and characterize electrophysiological severity. However, the relationship between preoperative NCS severity and postoperative recovery following carpal tunnel release remains uncertain. This study evaluated the association between preoperative electrophysiological severity and six-month outcomes following open carpal tunnel release.

Methods

This prospective observational departmental audit included 60 consecutive adult patients with clinically and electrophysiologically confirmed CTS who underwent primary open carpal tunnel release at Kulhudhuffushi Regional Hospital, Maldives. Patients were categorized as having mild, moderate, or severe CTS according to preoperative NCS findings based on the Bland grading system. Outcomes included the Boston Carpal Tunnel Questionnaire (BCTQ), visual analogue scale (VAS) pain score, grip strength, patient satisfaction, and postoperative complications. Outcomes were assessed up to six months following surgery. Associations involving ordinal NCS severity were assessed using Spearman's rank correlation, while the relationship between distal motor latency and six-month BCTQ score was assessed using Pearson's correlation.

Results

The mean age of the 60 patients was 51.8 ± 10.6 years, and 42 (70.0%) were women. Sixteen patients (26.7%) had mild CTS, 28 (46.7%) had moderate CTS, and 16 (26.7%) had severe CTS. BCTQ and VAS scores improved significantly following surgery across all three severity groups. Six-month BCTQ scores differed significantly among the mild, moderate, and severe groups (1.45 ± 0.41, 1.81 ± 0.58, and 2.50 ± 0.80, respectively; F = 12.33; p < 0.001). Six-month VAS scores (F = 6.59; p = 0.003) and grip strength (F = 6.06; p = 0.004) also differed significantly among severity groups. However, the magnitude of improvement in BCTQ score did not differ significantly among the groups (p = 0.399). Longer symptom duration was associated with greater electrophysiological severity (Spearman's ρ = 0.479; p < 0.001), while greater preoperative distal motor latency was associated with a higher six-month BCTQ score (Pearson's r = 0.520; p < 0.001). Fifty patients (83.3%) were satisfied or very satisfied with their surgical outcome.

Conclusions

Open carpal tunnel release resulted in substantial symptomatic improvement across all preoperative electrophysiological severity groups. Greater preoperative NCS severity was associated with poorer absolute six-month patient-reported and functional outcomes, although the magnitude of symptomatic improvement was not significantly different among severity groups. Prolonged distal motor latency was moderately associated with higher residual BCTQ scores at six months. These findings suggest that preoperative NCS findings may provide useful prognostic information when counselling patients regarding expected recovery after carpal tunnel release.

## Introduction

Carpal tunnel syndrome (CTS) is the most common entrapment neuropathy of the upper limb and results from the compression of the median nerve within the carpal tunnel [1,2]. It commonly presents with pain, numbness, paresthesia, and, in more advanced cases, weakness or thenar muscle dysfunction. CTS is common in the general population and represents an important cause of upper-limb symptoms and functional impairment [3]. Diagnosis is primarily based on clinical assessment, with electrodiagnostic testing used to confirm median nerve dysfunction, assess severity, and help exclude alternative or coexisting neuropathies [4].

Nerve conduction studies (NCS) provide objective measures of median nerve dysfunction and can be used to classify electrophysiological severity. The Bland neurophysiological grading system provides a structured method for categorizing CTS according to abnormalities in sensory and motor nerve conduction [5]. For patients with persistent or clinically significant symptoms despite appropriate conservative management, surgical decompression by division of the transverse carpal ligament is an established treatment option [6].

Clinical outcome after carpal tunnel release can be assessed using patient-reported and objective measures. The Boston Carpal Tunnel Questionnaire (BCTQ), originally developed by Levine et al., is a disease-specific instrument designed to assess symptom severity and functional status in patients with CTS [7]. Pain intensity can additionally be assessed using the visual analogue scale (VAS), a widely used method for quantifying subjective pain severity [8].

Although carpal tunnel release generally provides symptomatic and functional improvement, postoperative outcomes vary among patients. Several clinical factors have been investigated as potential predictors of outcome after surgery [9,10]. In particular, the relationship between preoperative electrophysiological severity and postoperative recovery remains clinically relevant because NCS is frequently performed before surgical treatment. Previous studies have examined whether preoperative NCS findings predict outcome after carpal tunnel decompression, with variable associations between electrophysiological severity and subsequent clinical recovery [11-13].

The present audit therefore aimed to evaluate the association between preoperative NCS severity and clinical outcomes following open carpal tunnel release at Kulhudhuffushi Regional Hospital. The primary objective was to compare six-month BCTQ outcomes among patients with mild, moderate, and severe electrophysiological CTS. Secondary objectives were to compare postoperative pain and grip strength among severity groups and to examine associations between selected preoperative electrophysiological parameters and six-month clinical outcomes.

## Materials and methods

Study design and setting

This prospective observational departmental audit was conducted in the Department of Orthopedics at Kulhudhuffushi Regional Hospital, Maldives. The study evaluated the association between preoperative nerve conduction study (NCS) severity and clinical and functional outcomes following primary open carpal tunnel release for carpal tunnel syndrome (CTS).

Study period and participants

A total of 60 consecutive adult patients with clinically and electrophysiologically confirmed CTS who underwent primary open carpal tunnel release were included.

Patients were eligible if they were aged >18 years, had a clinical diagnosis of CTS confirmed by NCS, underwent primary open carpal tunnel release, and completed the required postoperative follow-up.

Patients were excluded if they had undergone previous carpal tunnel release or had cervical radiculopathy, peripheral polyneuropathy, traumatic median nerve injury, inflammatory arthritis involving the wrist, pregnancy-related transient CTS, or incomplete follow-up data.

Patients underwent surgery between October 2024 and June 2025, with postoperative follow-up continuing for six months after surgery.

Preoperative clinical assessment

Demographic and clinical characteristics were recorded before surgery, including age, sex, symptom duration, relevant comorbidities, and clinical findings. Particular attention was given to diabetes mellitus, hypertension, hypothyroidism, nocturnal symptoms, thenar wasting, and provocative clinical tests.

Physical examination included Phalen's test, Tinel's sign, the assessment of thenar muscle wasting, sensory examination in the median nerve distribution, and motor assessment. Grip strength was measured using a handheld dynamometer and recorded in kilograms.

Nerve conduction study assessment

All patients underwent preoperative NCS performed using standardized neurophysiological techniques. Recorded electrophysiological parameters included distal motor latency, distal sensory latency, compound muscle action potential (CMAP) amplitude, and sensory nerve action potential (SNAP) amplitude.

Electrophysiological severity was classified according to the Bland neurophysiological grading system and grouped into three categories for analysis. Bland grades 1-2 were classified as mild CTS, grade 3 as moderate CTS, and grades 4-6 as severe CTS. Mild CTS therefore represented predominantly sensory conduction abnormalities, moderate CTS reflected the involvement of both sensory and motor conduction, and severe CTS represented advanced electrophysiological abnormalities, including marked motor latency prolongation, absent or markedly reduced sensory responses, or evidence of axonal loss. This grouping was applied consistently to all patients throughout the analysis. Representative motor and sensory nerve conduction findings are presented in Table 1 and Table 2, and corresponding median-ulnar comparison waveforms are shown in Figure 1.

**Table 1:** Representative motor nerve conduction study findings. Values represent findings from the representative preoperative nerve conduction study. NCV: nerve conduction velocity

Nerve	Side	Site	Latency (ms)	Duration (ms)	Amplitude (mV)	Area (mVms)	Segment	Distance (mm)	Interval (ms)	NCV (m/s)
Median	Left	Wrist	6.4	11.6	8.9	27.5	Wrist	-	6.4	-
Median	Left	Elbow	10.2	11.7	7.8	24.8	Wrist-elbow	220	3.9	56.8
Median	Right	Wrist	6.6	11.6	7.9	23.3	Wrist	-	6.6	-
Median	Right	Elbow	10.9	11.8	7.3	22.6	Wrist-elbow	220	4.4	50.6
Ulnar	Left	Wrist	2.4	10.2	16.7	47.2	Wrist	-	2.4	-
Ulnar	Left	Elbow	6.5	10.3	16.3	42.7	Wrist-elbow	240	4	59.4
Ulnar	Right	Wrist	2.6	9.8	13.5	35	Wrist	-	2.6	-
Ulnar	Right	Elbow	6.6	10	12.6	32.2	Wrist-elbow	240	4	59.4

**Table 2:** Representative sensory nerve conduction study findings. The left median sensory response was absent. The right median sensory study demonstrated prolonged latency and reduced conduction velocity compared with the ulnar sensory responses. NCV: nerve conduction velocity

Nerve	Side	Site	Latency 1 (ms)	Latency 2 (ms)	Amplitude (µV)	Area (µVms)	Segment	Distance (mm)	Interval (ms)	NCV (m/s)
Median	Left	Wrist	-	-	-	-	Wrist	-	-	-
Median	Right	Wrist	6	7	11.3	0.2	Wrist	130	6	21.6
Ulnar	Left	Wrist	1.9	2.5	29.3	1.4	Wrist	110	1.9	59.1
Ulnar	Right	Wrist	2	2.5	27.3	0.6	Wrist	110	2	55.6

**Figure 1:**
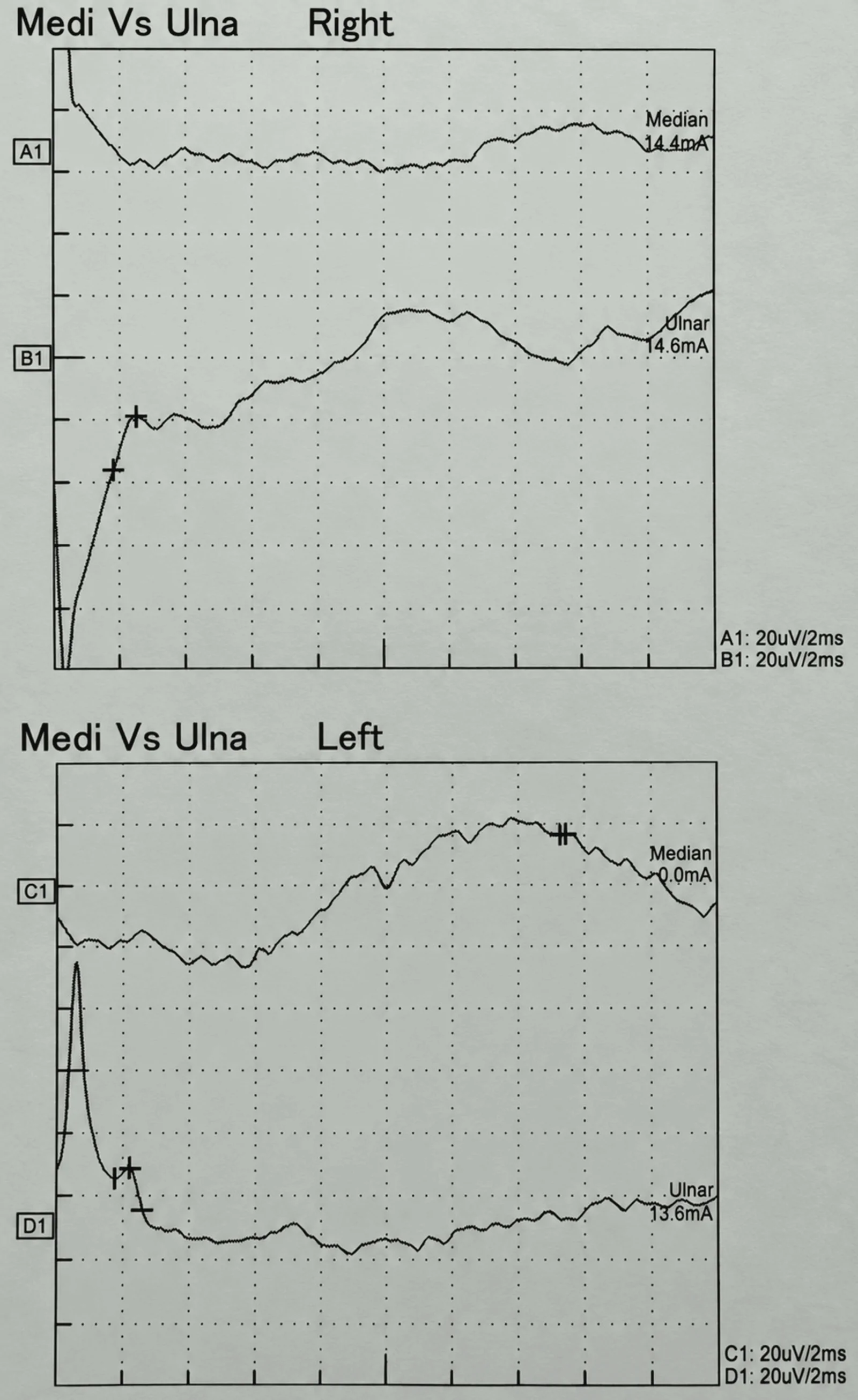
Representative median-ulnar comparison waveforms in carpal tunnel syndrome. Representative median-ulnar comparison nerve conduction study waveforms demonstrating abnormal median nerve conduction relative to the ulnar nerve. The findings are consistent with median neuropathy at the wrist and illustrate the electrophysiological abnormalities used in the assessment of carpal tunnel syndrome severity.

Surgical procedure

All patients underwent standard open carpal tunnel release. Following sterile preparation and draping, a longitudinal palmar incision was made, and the transverse carpal ligament was identified and completely divided under direct visualization to decompress the median nerve.

Hemostasis was achieved before wound closure, and the skin was closed with interrupted nylon sutures. Patients were encouraged to begin early finger mobilization.

Postoperatively, patients were prescribed oral etoricoxib 60 mg twice daily for three to five days for analgesia, together with routine wound-care instructions. No separate rescue analgesic was routinely prescribed. The postoperative analgesic regimen was standardized for the study patients.

Sutures were removed approximately 10-14 days after surgery. Representative intraoperative photographs of the open carpal tunnel release technique are shown in Figure 2.

**Figure 2:**
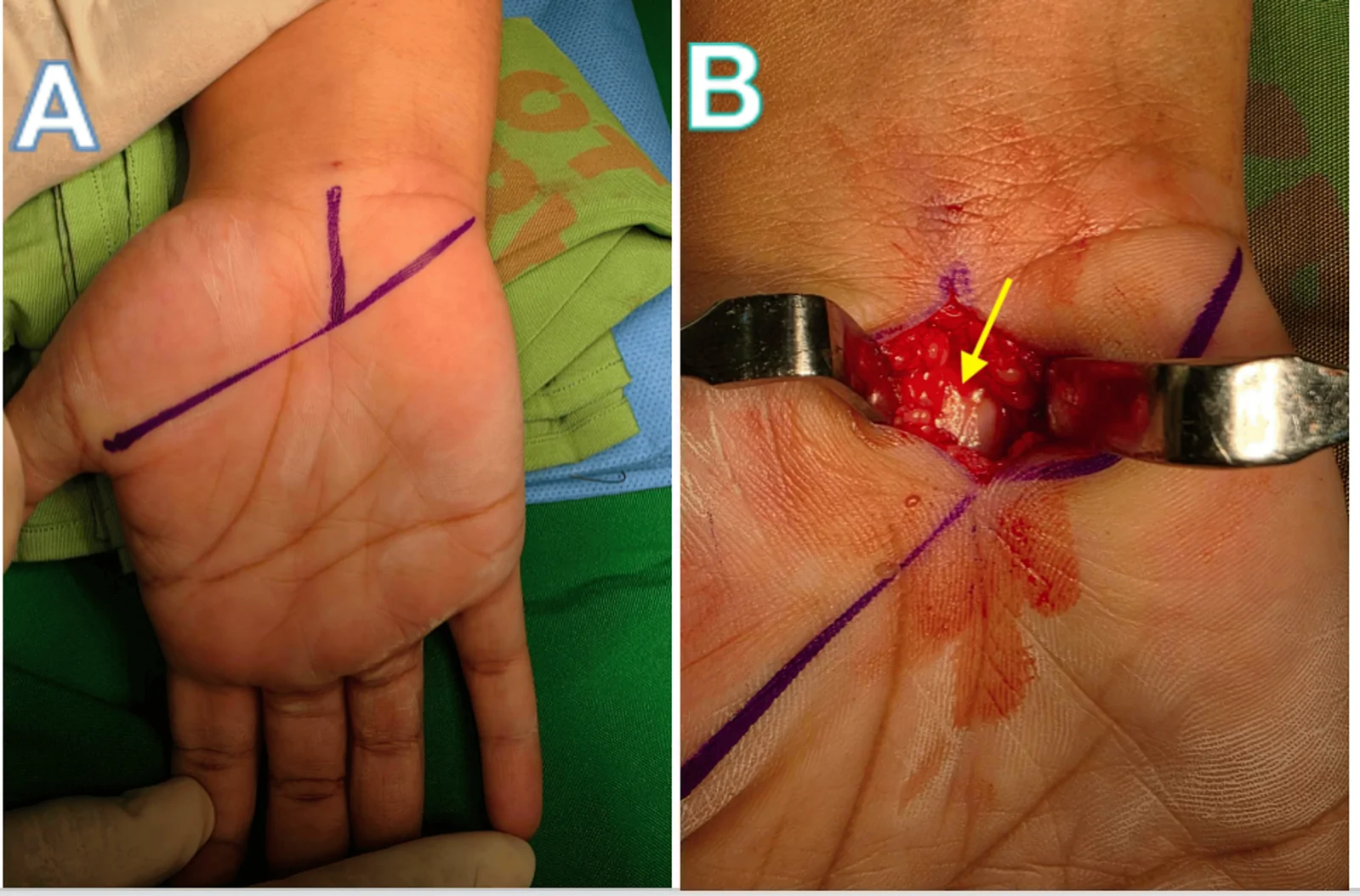
Operative technique for open carpal tunnel release. Representative intraoperative photographs demonstrating open carpal tunnel release. (A) Preoperative marking of the planned palmar incision over the carpal tunnel. (B) Intraoperative view following decompression, demonstrating the exposed median nerve (yellow arrow) within the carpal tunnel.

Follow-up and outcome assessment

Patients underwent routine postoperative clinical follow-up, with the principal outcome assessment for this audit performed at six months. BCTQ score, VAS pain score, grip strength, patient satisfaction, and postoperative complications at six months were used for the present analysis.

Patient-reported outcomes were assessed using the Boston Carpal Tunnel Questionnaire (BCTQ), originally described by Levine et al. [7]. The BCTQ is a disease-specific instrument for evaluating symptom severity and functional status, with higher scores indicating greater symptom burden or functional limitation. The BCTQ was administered in English. For patients who required language assistance, individual questions were verbally explained or translated into Dhivehi by local staff nurses or a translator. A formally validated and culturally adapted Dhivehi version of the BCTQ was not used.

The VAS was used to assess overall CTS-related hand/wrist pain rather than a specific pain domain such as pain at rest, pain during activity, or nocturnal pain. Pain intensity was recorded using a 10 cm horizontal VAS anchored by "no pain" at 0 and "worst imaginable pain" at 10. Patients marked the point on the line that best represented their overall CTS-related hand/wrist pain, and the distance from the zero anchor to the patient's mark was used to determine the pain score [8]. Grip strength was measured in kilograms using a handheld dynamometer. Patient satisfaction and postoperative complications were also recorded.

Patient satisfaction at final follow-up was categorized as very satisfied, satisfied, fair, or poor. Recorded postoperative complications included superficial wound infection, persistent numbness, scar tenderness, pillar pain, and recurrence.

Statistical analysis

Data were analyzed using IBM SPSS Statistics version 26 (IBM Corp., Armonk, NY). Continuous variables were summarized as mean ± standard deviation (SD), while categorical variables were presented as frequencies and percentages. All statistical tests were two-sided, and a p-value of <0.05 was considered statistically significant.

Within-group changes in BCTQ score, VAS pain score, and grip strength between the preoperative assessment and six-month follow-up were evaluated using paired-samples t-tests. Differences in six-month BCTQ score, VAS pain score, and grip strength among the mild, moderate, and severe electrophysiological severity groups were assessed using one-way analysis of variance (ANOVA). Changes from baseline were also calculated and compared among the three severity groups using one-way ANOVA to determine whether the magnitude of postoperative improvement differed according to preoperative electrophysiological severity.

Because NCS severity was an ordinal variable consisting of mild, moderate, and severe categories, its association with symptom duration was assessed using Spearman's rank correlation coefficient (ρ). The association between preoperative distal motor latency and six-month BCTQ score was assessed using Pearson's correlation coefficient (r), treating both variables as continuous measures.

Ethical considerations

The project was conducted within the quality improvement and patient safety framework of the Quality Improvement Department of Kulhudhuffushi Regional Hospital (approval number: KRH/QID/AUD/2026/004). Written informed consent was obtained from participants, and patient information was anonymized for analysis and reporting. Patient confidentiality was maintained throughout the project.

As the project was conducted as a departmental clinical audit/quality-improvement activity using routinely collected clinical and electrophysiological information, formal national ethics review was not required according to the institutional audit and quality-improvement process.

## Results

Patient characteristics

A total of 60 patients who underwent primary open carpal tunnel release were included in the analysis, and all completed six-month follow-up. The mean age was 51.8 ± 10.6 years (range: 32-73 years), and 42 patients (70.0%) were women. Diabetes mellitus was present in 18 patients (30.0%), hypertension in 15 (25.0%), and hypothyroidism in four (6.7%).

The mean duration of symptoms before surgery was 14.2 ± 7.5 months. Ten patients (16.7%) had symptoms for less than six months, 22 (36.7%) for six to 12 months, and 28 (46.7%) for more than 12 months.

Nocturnal symptoms were present in 56 patients (93.3%), Phalen's test was positive in 54 (90.0%), Tinel's sign was positive in 49 (81.7%), and thenar wasting was documented in 11 (18.3%). Baseline demographic and clinical characteristics are summarized in Table 3.

**Table 3:** Baseline demographic and clinical characteristics of the study population (n = 60). Values are presented as mean ± standard deviation (SD) for continuous variables and number (percentage) for categorical variables. n: number of patients

Characteristic	Value
Age, years, mean ± SD	51.8 ± 10.6
Female sex, n (%)	42 (70.0)
Male sex, n (%)	18 (30.0)
Diabetes mellitus, n (%)	18 (30.0)
Hypertension, n (%)	15 (25.0)
Hypothyroidism, n (%)	4 (6.7)
Symptom duration, months, mean ± SD	14.2 ± 7.5
Symptom duration <6 months, n (%)	10 (16.7)
Symptom duration 6-12 months, n (%)	22 (36.7)
Symptom duration >12 months, n (%)	28 (46.7)
Nocturnal symptoms, n (%)	56 (93.3)
Positive Phalen's test, n (%)	54 (90.0)
Positive Tinel's sign, n (%)	49 (81.7)
Thenar wasting, n (%)	11 (18.3)

Preoperative nerve conduction study findings

According to electrophysiological severity, 16 patients (26.7%) had mild CTS, 28 (46.7%) had moderate CTS, and 16 (26.7%) had severe CTS. Moderate CTS was therefore the most common electrophysiological category (Figure 3).

**Figure 3:**
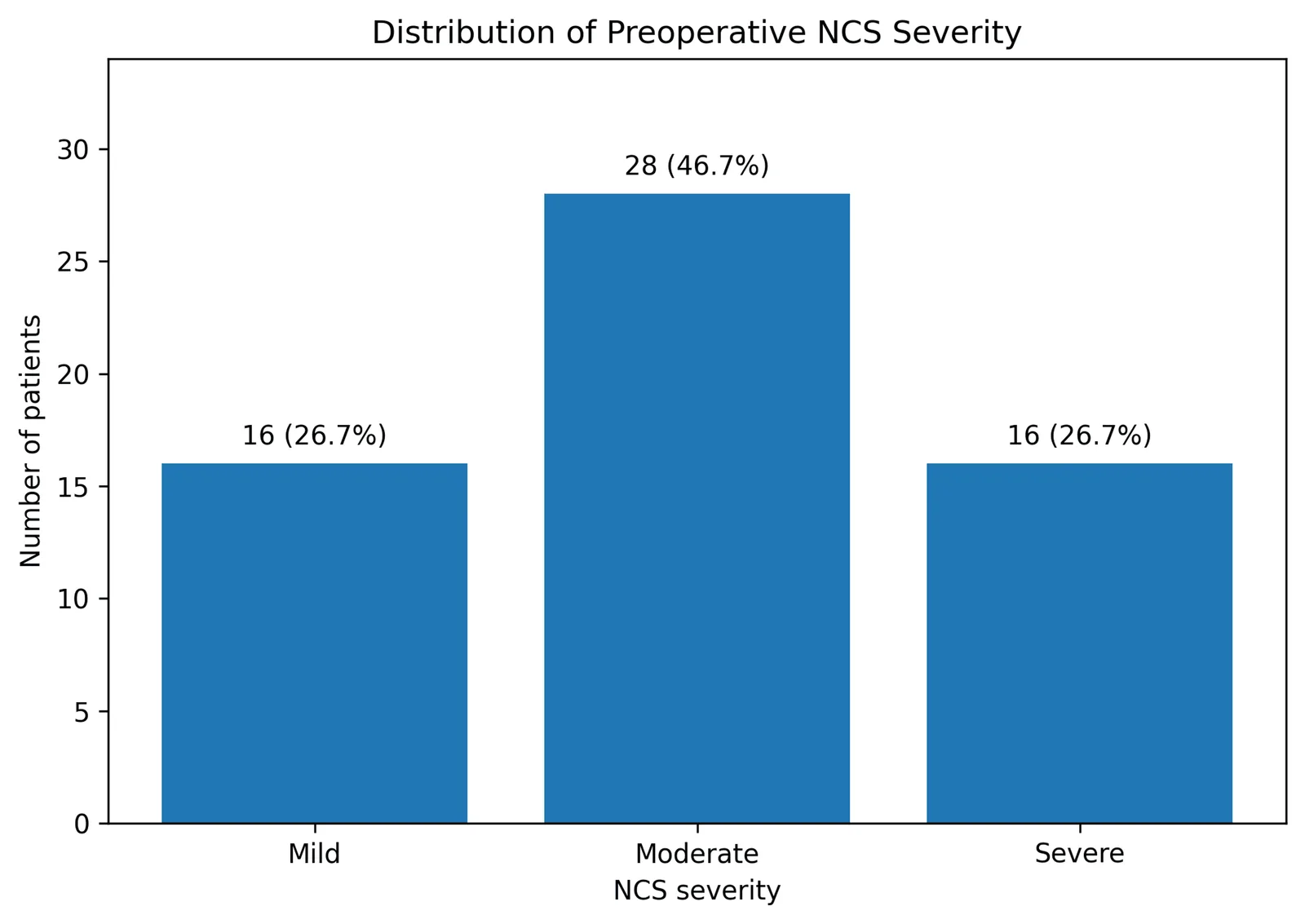
Distribution of preoperative nerve conduction study severity. Preoperative nerve conduction study (NCS) findings classified 16 patients (26.7%) as having mild carpal tunnel syndrome (CTS), 28 (46.7%) as having moderate CTS, and 16 (26.7%) as having severe CTS according to the electrophysiological severity classification used in the study.

Progressive deterioration in nerve conduction parameters was observed with increasing severity. Mean distal motor latency increased from 4.3 ± 0.5 ms in the mild group to 5.8 ± 0.7 ms in the moderate group and 7.6 ± 1.1 ms in the severe group. Conversely, CMAP and SNAP amplitudes decreased with increasing electrophysiological severity. Preoperative NCS parameters according to electrophysiological severity are presented in Table 4.

**Table 4:** Preoperative nerve conduction study parameters according to electrophysiological severity. Values are presented as mean ± standard deviation (SD). Between-group comparisons were performed using one-way analysis of variance (ANOVA). A p-value of <0.05 was considered statistically significant. CMAP, compound muscle action potential; SNAP, sensory nerve action potential; NCS, nerve conduction study

NCS parameter	Mild (n = 16)	Moderate (n = 28)	Severe (n = 16)	F statistic	P-value
Distal motor latency (ms)	4.30 ± 0.50	5.80 ± 0.70	7.60 ± 1.10	71.02	<0.001
Sensory latency (ms)	3.60 ± 0.40	4.80 ± 0.50	6.10 ± 0.80	76.03	<0.001
CMAP amplitude (mV)	8.40 ± 1.20	6.90 ± 1.30	4.54 ± 1.38	36.29	<0.001
SNAP amplitude (µV)	18.50 ± 4.10	12.80 ± 3.70	6.20 ± 2.40	48.82	<0.001

Six-month clinical outcomes

Substantial improvement in symptom scores and pain was observed at six months in all three electrophysiological severity groups. Mean BCTQ score decreased from 3.50 ± 0.40 to 1.45 ± 0.41 in the mild group, from 3.90 ± 0.50 to 1.81 ± 0.58 in the moderate group, and from 4.26 ± 0.52 to 2.50 ± 0.80 in the severe group. Within-group improvement in BCTQ score was statistically significant in all three severity groups (all p < 0.001).

VAS pain scores also improved significantly in all groups (all p < 0.001). Grip strength increased from 19.50 ± 5.20 kg to 24.60 ± 5.80 kg in patients with mild CTS (p = 0.018) and from 18.10 ± 4.70 kg to 22.80 ± 5.10 kg in those with moderate CTS (p < 0.001). In the severe group, mean grip strength increased from 15.20 ± 4.30 kg to 18.40 ± 4.90 kg; however, this within-group improvement did not reach conventional statistical significance (p = 0.053).

At six months, postoperative BCTQ scores differed significantly among the three NCS severity groups (one-way ANOVA, F = 12.33 and p < 0.001), with higher residual symptom scores in patients with severe CTS. Postoperative VAS scores (F = 6.59; p = 0.003) and grip strength (F = 6.06; p = 0.004) also differed significantly across severity groups.

Importantly, although absolute six-month outcomes were poorer in patients with greater preoperative NCS severity, the magnitude of BCTQ improvement did not differ significantly among the three groups (F = 0.93; p = 0.399). Similarly, between-group differences in VAS reduction (F = 0.44; p = 0.649) and grip-strength improvement (F = 0.37; p = 0.690) were not statistically significant. Preoperative and six-month clinical outcomes according to electrophysiological severity are summarized in Table 5. Changes in BCTQ scores according to preoperative NCS severity are illustrated in Figure 4.

**Table 5:** Preoperative and six-month outcomes according to electrophysiological severity. Values are presented as mean ± standard deviation (SD). Improvement in BCTQ and VAS represents the reduction from the preoperative score to the six-month score, whereas improvement in grip strength represents the increase from the preoperative value to the six-month value. Between-group comparisons were performed using one-way analysis of variance (ANOVA), with the corresponding F statistics and p-values reported. A p-value of <0.05 was considered statistically significant. BCTQ, Boston Carpal Tunnel Questionnaire; VAS, visual analogue scale

Clinical outcome	Mild (n = 16)	Moderate (n = 28)	Severe (n = 16)	F statistic	P-value
BCTQ, preoperative	3.50 ± 0.40	3.90 ± 0.50	4.26 ± 0.52	-	-
BCTQ, six months	1.45 ± 0.41	1.81 ± 0.58	2.50 ± 0.80	12.33	<0.001
BCTQ improvement	2.05 ± 0.62	2.09 ± 0.75	1.76 ± 0.99	0.93	0.399
VAS, preoperative	7.10 ± 1.10	7.60 ± 1.00	8.20 ± 0.90	-	-
VAS, six months	1.90 ± 1.00	2.32 ± 1.16	3.40 ± 1.50	6.59	0.003
VAS reduction	5.20 ± 1.37	5.28 ± 1.58	4.80 ± 2.11	0.44	0.649
Grip strength (kg), preoperative	19.50 ± 5.20	18.10 ± 4.70	15.20 ± 4.30	-	-
Grip strength (kg), six months	24.60 ± 5.80	22.80 ± 5.10	18.40 ± 4.90	6.06	0.004
Grip-strength improvement (kg)	5.10 ± 7.65	4.70 ± 6.44	3.20 ± 6.08	0.37	0.69

**Figure 4:**
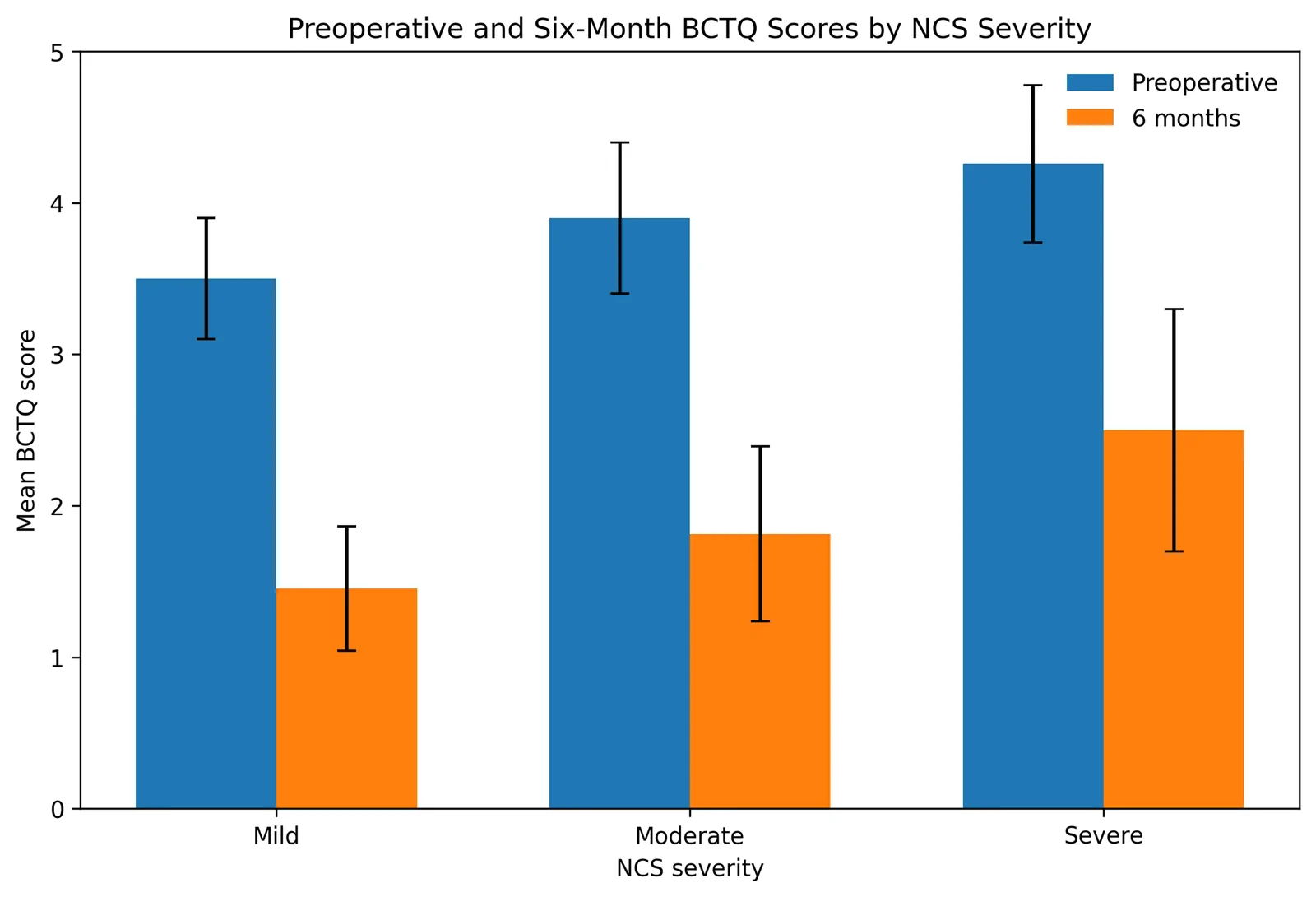
Preoperative and six-month BCTQ scores according to nerve conduction study severity. Mean Boston Carpal Tunnel Questionnaire (BCTQ) scores before surgery and at six months following open carpal tunnel release in patients with mild, moderate, and severe electrophysiological CTS. Error bars represent standard deviations. BCTQ scores improved following surgery in all three groups. At six months, scores differed significantly among the severity groups (one-way ANOVA, F = 12.33 and p < 0.001), with higher residual scores observed in the severe group. Higher BCTQ scores indicate greater symptom burden and functional limitation. NCS, nerve conduction study; CTS, carpal tunnel syndrome; ANOVA, analysis of variance

Association between preoperative NCS findings and postoperative outcomes

Because electrophysiological severity was an ordinal variable, Spearman's rank correlation was used to evaluate associations involving the mild, moderate, and severe NCS categories. Longer preoperative symptom duration was moderately associated with greater electrophysiological severity (Spearman's ρ = 0.479; p < 0.001).

Distal motor latency, analyzed as a continuous electrophysiological variable, demonstrated a moderate positive correlation with the six-month BCTQ score (Pearson's r = 0.520; p < 0.001). Thus, the greater prolongation of preoperative distal motor latency was associated with higher residual BCTQ scores at six months. The associations between preoperative electrophysiological variables and postoperative outcomes are summarized in Table 6. The association between preoperative distal motor latency and six-month BCTQ score is illustrated in Figure 5.

**Figure 5:**
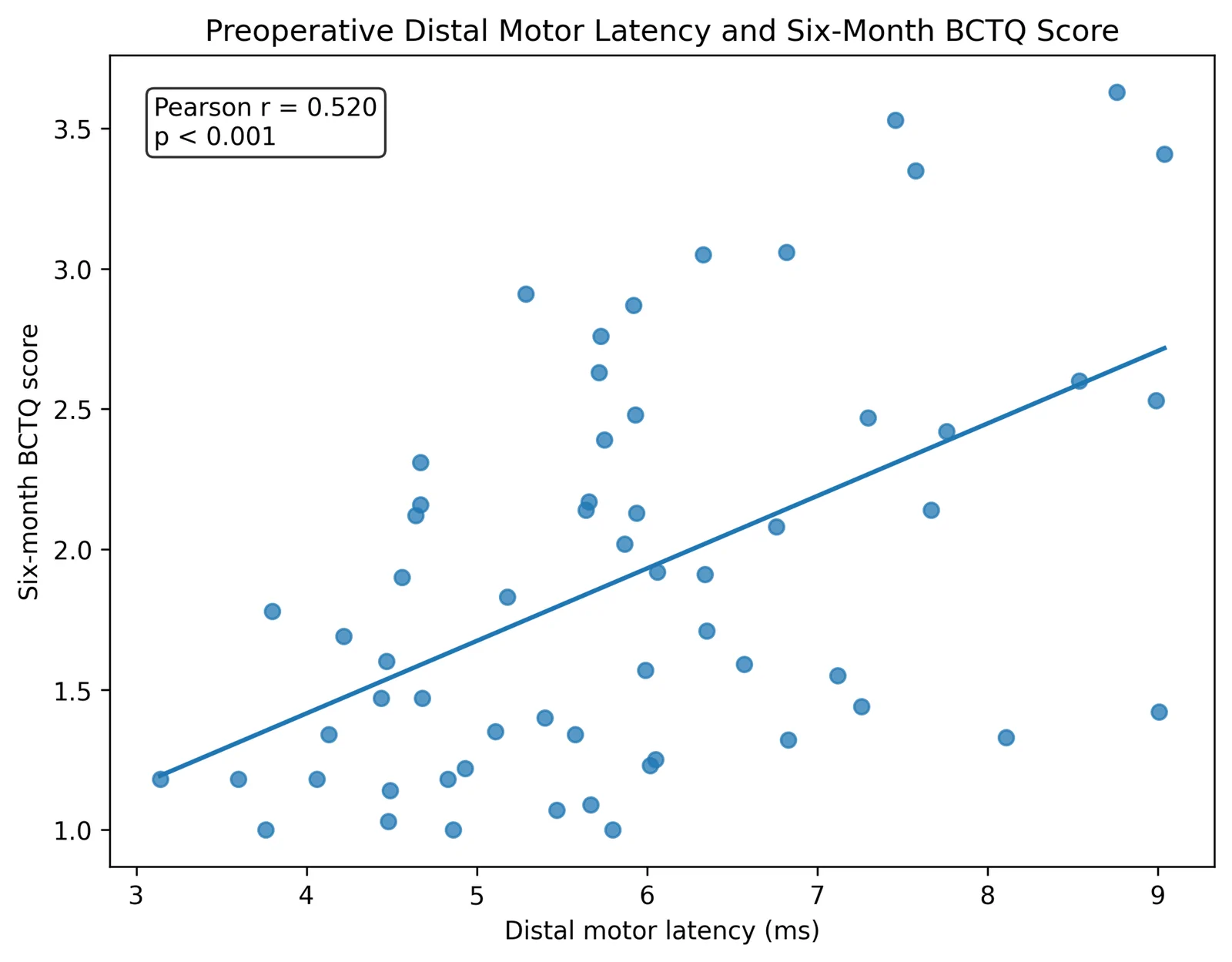
Association between preoperative distal motor latency and six-month BCTQ score. Scatterplot demonstrating the relationship between preoperative distal motor latency and Boston Carpal Tunnel Questionnaire (BCTQ) score at six months following open carpal tunnel release. The solid line represents the fitted linear regression line. A moderate positive correlation was observed (Pearson's r = 0.520; p < 0.001), indicating that longer preoperative distal motor latency was associated with higher residual BCTQ scores at six months.

**Table 6:** Associations between preoperative electrophysiological variables and postoperative outcomes. Spearman's rank correlation coefficient (ρ) was used to assess the association between symptom duration and ordinal NCS severity. Pearson's correlation coefficient (r) was used to assess the association between preoperative distal motor latency and six-month BCTQ score. A p-value of <0.05 was considered statistically significant. BCTQ, Boston Carpal Tunnel Questionnaire; NCS, nerve conduction study

Variables	Statistical test	Correlation coefficient	P-value
Symptom duration versus NCS severity	Spearman's rank correlation	ρ = 0.479	<0.001
Distal motor latency versus six-month BCTQ score	Pearson's correlation	r = 0.520	<0.001

Patient satisfaction and complications

At six months, 28 patients (46.7%) reported being very satisfied, and 22 (36.7%) were satisfied with the outcome of surgery. Eight patients (13.3%) reported fair satisfaction, and two (3.3%) reported poor satisfaction. Therefore, 50 of 60 patients (83.3%) were either satisfied or very satisfied following open carpal tunnel release.

Postoperative complications were uncommon. Superficial wound infection occurred in two patients (3.3%), persistent numbness in five (8.3%), scar tenderness in four (6.7%), and pillar pain in three (5.0%). No major complication was recorded in the dataset.

Persistent numbness was documented in five patients (8.3%) at six-month follow-up. All five cases occurred in the moderate electrophysiological severity group (5/28, 17.9%), whereas no persistent numbness was recorded in the mild (0/16) or severe (0/16) groups. Given the small number of events and zero counts in two severity categories, an exact test was used; the association between preoperative NCS severity and persistent numbness did not reach statistical significance (Fisher-Freeman-Halton exact test, p = 0.074).

Overall, open carpal tunnel release was associated with substantial improvement in patient-reported symptoms and pain across all electrophysiological severity groups. Patients with severe preoperative NCS abnormalities had poorer absolute six-month BCTQ, VAS, and grip-strength outcomes; however, the magnitude of improvement in BCTQ and VAS scores was not significantly different among severity groups.

## Discussion

The present audit demonstrated significant clinical improvement following open carpal tunnel release across all three preoperative electrophysiological severity groups. BCTQ scores, pain scores, and grip strength improved substantially by six months. However, patients with severe preoperative NCS abnormalities had higher residual BCTQ and pain scores and lower grip strength at six months than those with mild or moderate disease. Importantly, the magnitude of BCTQ improvement did not differ significantly among the three groups, suggesting that substantial symptomatic and functional improvement can occur even in patients with severe electrophysiological CTS.

These findings are consistent with the broader literature evaluating the role of electrodiagnostic testing in CTS. A systematic review by Osiak et al. highlighted the value of electrodiagnostic studies in diagnosis, severity assessment, and surgical decision-making while also demonstrating that the relationship between electrodiagnostic severity and postoperative outcome is not uniform across studies [14]. Similarly, a systematic review focusing on severe CTS found that patients with advanced disease can still achieve meaningful symptomatic and functional improvement following carpal tunnel release, although recovery may be incomplete in some patients [15].

The less favorable absolute six-month outcomes observed in the severe group may reflect more advanced median nerve dysfunction before decompression. In severe CTS, prolonged compression may be associated with marked conduction abnormalities and axonal involvement, potentially limiting or delaying neurological recovery. Sasaki et al. reported that electrophysiological parameters may have prognostic value in patients with severe CTS and identified nerve conduction characteristics associated with postoperative improvement [16]. Nevertheless, the significant improvement observed across all severity groups in the present audit supports the view that severe electrophysiological abnormalities alone should not be interpreted as evidence that surgical decompression will provide little clinical benefit.

Postoperative recovery after carpal tunnel release is multifactorial. Factors other than NCS severity, including symptom duration, baseline functional impairment, age, comorbidities, and other patient characteristics, may influence outcome. Previous work has demonstrated that several independent variables can affect recovery after carpal tunnel release [17], while reviews of the literature have similarly concluded that no single preoperative variable consistently predicts postoperative outcome in all patients [18]. This may partly explain why the severe group in the present audit had worse absolute six-month scores despite demonstrating substantial improvement from baseline.

Comorbid conditions may also influence postoperative recovery. Diabetes mellitus is particularly relevant because it may coexist with peripheral neuropathy and potentially affect nerve recovery. Previous studies comparing diabetic and nondiabetic patients have nevertheless demonstrated improvement after carpal tunnel release in both groups, although differences in the degree or pattern of recovery have been reported [19,20]. In the present audit, diabetes was present in a proportion of patients, but the sample size was not designed to permit adequately powered subgroup analysis according to diabetic status.

Patient satisfaction was high, with 83.3% of patients reporting that they were satisfied or very satisfied at six months. This finding is consistent with the generally favorable patient-reported outcomes reported after carpal tunnel release. Long-term studies have also demonstrated sustained patient-centered benefits following surgical decompression [21]. The low complication rate observed in the present audit further supports open carpal tunnel release as an effective procedure in this regional hospital setting.

The observed relationship between electrophysiological severity and postoperative patient-reported outcome is also consistent with studies examining the association between electrodiagnostic findings and BCTQ scores. Koo et al. reported a relationship between electrodiagnostic severity and BCTQ measures in surgically treated patients with CTS [22]. In the present audit, preoperative distal motor latency showed a moderate positive correlation with six-month BCTQ score, indicating that greater preoperative motor conduction abnormality was associated with greater residual symptom burden or functional limitation at follow-up. Symptom duration was also positively associated with NCS severity, suggesting that patients with longer-standing symptoms tended to present with more advanced electrophysiological abnormalities.

Strengths and limitations

A strength of this audit is the use of standardized electrophysiological severity classification, together with patient-reported and objective clinical outcome measures. The inclusion of BCTQ scores, pain assessment, grip strength, patient satisfaction, and complications provided a multidimensional assessment of postoperative recovery. The audit also provides clinical outcome data from a regional island hospital setting, where published evidence on CTS treatment is limited.

Several limitations should be acknowledged. First, the sample size was relatively small, particularly after stratification into mild, moderate, and severe electrophysiological severity groups. Second, the six-month follow-up period may not capture the full extent of neurological recovery, particularly in patients with severe CTS, in whom recovery may be prolonged. Third, this was a single-institution audit, which may limit the generalizability of the findings to other clinical settings and populations. Potential confounding factors, including age, diabetes mellitus, symptom duration, and other comorbidities, were not adjusted for using multivariable analysis. In addition, the BCTQ was administered in English, with verbal explanation or translation into Dhivehi when required. As a formally validated and culturally adapted Dhivehi version was not used, linguistic and cultural differences may have influenced patients' interpretation of individual questionnaire items, introducing potential measurement bias. The BCTQ was also analyzed as an overall score rather than as separate Symptom Severity Scale and Functional Status Scale components; consequently, it was not possible to determine whether differences in postoperative BCTQ scores were predominantly attributable to residual symptoms or functional limitations. Finally, postoperative NCS was not routinely performed; therefore, this audit evaluated clinical and functional recovery rather than electrophysiological recovery following decompression.

## Conclusions

Open carpal tunnel release resulted in substantial symptomatic improvement across all levels of preoperative electrophysiological severity. Patients with severe nerve conduction study (NCS) abnormalities had higher residual BCTQ and pain scores and lower grip strength at six months; however, the magnitude of improvement in BCTQ score, pain, and grip strength did not differ significantly among the mild, moderate, and severe groups. Longer symptom duration was associated with greater electrophysiological severity, while prolonged distal motor latency was associated with higher residual BCTQ scores at six months.

These findings suggest that preoperative NCS may provide useful prognostic information when counselling patients about expected recovery but should not be used as the sole predictor of surgical outcome. Importantly, patients with severe electrophysiological CTS can still achieve meaningful improvement following open carpal tunnel release.

## References

[1] Padua L, Coraci D, Erra C (2016). Carpal tunnel syndrome: clinical features, diagnosis, and management. Lancet Neurol.

[2] Ibrahim I, Khan WS, Goddard N, Smitham P (2012). Carpal tunnel syndrome: a review of the recent literature. Open Orthop J.

[3] Atroshi I, Gummesson C, Johnsson R, Ornstein E, Ranstam J, Rosén I (1999). Prevalence of carpal tunnel syndrome in a general population. JAMA.

[4] Wipperman J, Goerl K (2016). Carpal tunnel syndrome: diagnosis and management. Am Fam Physician.

[5] Bland JD (2000). A neurophysiological grading scale for carpal tunnel syndrome. Muscle Nerve.

[6] Scholten RJ, Mink van der Molen A, Uitdehaag BM, Bouter LM, de Vet HC (2007). Surgical treatment options for carpal tunnel syndrome. Cochrane Database Syst Rev.

[7] Levine DW, Simmons BP, Koris MJ, Daltroy LH, Hohl GG, Fossel AH, Katz JN (1993). A self-administered questionnaire for the assessment of severity of symptoms and functional status in carpal tunnel syndrome. J Bone Joint Surg Am.

[8] Huskisson EC (1974). Measurement of pain. Lancet.

[9] Katz JN, Losina E, Amick BC 3rd, Fossel AH, Bessette L, Keller RB (2001). Predictors of outcomes of carpal tunnel release. Arthritis Rheum.

[10] Conzen C, Conzen M, Rübsamen N, Mikolajczyk R (2016). Predictors of the patient-centered outcomes of surgical carpal tunnel release - a prospective cohort study. BMC Musculoskelet Disord.

[11] Bland JD (2001). Do nerve conduction studies predict the outcome of carpal tunnel decompression?. Muscle Nerve.

[12] Longstaff L, Milner RH, O'Sullivan S, Fawcett P (2001). Carpal tunnel syndrome: the correlation between outcome, symptoms and nerve conduction study findings. J Hand Surg Br.

[13] Rivlin M, Kachooei AR, Wang ML, Ilyas AM (2018). Electrodiagnostic grade and carpal tunnel release outcomes: a prospective analysis. J Hand Surg Am.

[14] Osiak K, Mazurek A, Pękala P, Koziej M, Walocha JA, Pasternak A (2021). Electrodiagnostic studies in the surgical treatment of carpal tunnel syndrome-a systematic review. J Clin Med.

[15] Meyers A, Annunziata MJ, Rampazzo A, Bassiri Gharb B (2023). A systematic review of the outcomes of carpal ligament release in severe carpal tunnel syndrome. J Hand Surg Am.

[16] Sasaki Y, Terao T, Saito E (2020). Clinical predictors of surgical outcomes of severe carpal tunnel syndrome patients: utility of palmar stimulation in a nerve conduction study. BMC Musculoskelet Disord.

[17] Watchmaker JD, Watchmaker GP (2018). Independent variables affecting outcome of carpal tunnel release surgery. Hand (N Y).

[18] Turner A, Kimble F, Gulyás K, Ball J (2010). Can the outcome of open carpal tunnel release be predicted?: a review of the literature. ANZ J Surg.

[19] Ozkul Y, Sabuncu T, Kocabey Y, Nazligul Y (2002). Outcomes of carpal tunnel release in diabetic and non-diabetic patients. Acta Neurol Scand.

[20] Zyluk A, Puchalski P (2013). A comparison of outcomes of carpal tunnel release in diabetic and non-diabetic patients. J Hand Surg Eur Vol.

[21] Dawod MS, Alswerki MN, Alelaumi AF (2025). Long-term patient-centered outcomes following carpal tunnel release surgery: a 10-year follow-up. Langenbecks Arch Surg.

[22] Koo JH, Bae JY, Lee K, Park HS (2023). Correlation between electrodiagnostic severity and Boston carpal tunnel questionnaire in surgically treated carpal tunnel syndrome patients. Acta Orthop Traumatol Turc.

